# Conversational Agents in Health Care: Scoping Review of Their Behavior Change Techniques and Underpinning Theory

**DOI:** 10.2196/39243

**Published:** 2022-10-03

**Authors:** Laura Martinengo, Ahmad Ishqi Jabir, Westin Wei Tin Goh, Nicholas Yong Wai Lo, Moon-Ho Ringo Ho, Tobias Kowatsch, Rifat Atun, Susan Michie, Lorainne Tudor Car

**Affiliations:** 1 Lee Kong Chian School of Medicine Nanyang Technological University Singapore Singapore Singapore; 2 School of Social Sciences Nanyang Technological University Singapore Singapore Singapore; 3 Institute for Implementation Science in Health Care University of Zurich Zurich Switzerland; 4 School of Medicine University of St.Gallen St. Gallen Switzerland; 5 Centre for Digital Health Interventions Department of Management, Technology, and Economics ETH Zurich Zurich Switzerland; 6 Future Health Technologies Programme Campus for Research Excellence and Technological Enterprise Singapore-ETH Centre Singapore Singapore; 7 Department of Global Health and Population Harvard T.H. Chan School of Public Health Harvard University Cambridge, MA United States; 8 Department of Global Health and Social Medicine Harvard Medical School Harvard University Cambridge, MA United States; 9 Health Systems Innovation Lab Harvard T.H. Chan School of Public Health Harvard University Cambridge, MA United States; 10 Department of Health Policy and Management Harvard T.H. Chan School of Public Health Harvard University Cambridge, MA United States; 11 UCL Centre for Behaviour Change University College London London United Kingdom; 12 Department of Primary Care and Public Health School of Public Health Imperial College London London United Kingdom

**Keywords:** behavior change, behavior change techniques, conversational agent, chatbot, mHealth

## Abstract

**Background:**

Conversational agents (CAs) are increasingly used in health care to deliver behavior change interventions. Their evaluation often includes categorizing the behavior change techniques (BCTs) using a classification system of which the BCT Taxonomy v1 (BCTTv1) is one of the most common. Previous studies have presented descriptive summaries of behavior change interventions delivered by CAs, but no in-depth study reporting the use of BCTs in these interventions has been published to date.

**Objective:**

This review aims to describe behavior change interventions delivered by CAs and to identify the BCTs and theories guiding their design.

**Methods:**

We searched PubMed, Embase, Cochrane’s Central Register of Controlled Trials, and the first 10 pages of Google and Google Scholar in April 2021. We included primary, experimental studies evaluating a behavior change intervention delivered by a CA. BCTs coding followed the BCTTv1. Two independent reviewers selected the studies and extracted the data. Descriptive analysis and frequent itemset mining to identify BCT clusters were performed.

**Results:**

We included 47 studies reporting on mental health (n=19, 40%), chronic disorders (n=14, 30%), and lifestyle change (n=14, 30%) interventions. There were 20/47 embodied CAs (43%) and 27/47 CAs (57%) represented a female character. Most CAs were rule based (34/47, 72%). Experimental interventions included 63 BCTs, (mean 9 BCTs; range 2-21 BCTs), while comparisons included 32 BCTs (mean 2 BCTs; range 2-17 BCTs). Most interventions included BCTs 4.1 “Instruction on how to perform a behavior” (34/47, 72%), 3.3 “Social support” (emotional; 27/47, 57%), and 1.2 “Problem solving” (24/47, 51%). A total of 12/47 studies (26%) were informed by a behavior change theory, mainly the Transtheoretical Model and the Social Cognitive Theory. Studies using the same behavior change theory included different BCTs.

**Conclusions:**

There is a need for the more explicit use of behavior change theories and improved reporting of BCTs in CA interventions to enhance the analysis of intervention effectiveness and improve the reproducibility of research.

## Introduction

Conversational agents (CAs), or chatbots, are computer programs that simulate conversations with humans [1]. Although the first CAs were developed in the mid-1960s, it was not until the early 2000s that their availability and popularity markedly increased [2]. CAs can be used to automate a variety of tasks, such as the provision of news or weather forecasts and the facilitation of web-based shopping [3]. CAs may be deployed as stand-alone apps or websites, integrated into multifunctional apps, or included in messaging apps such as Telegram, Facebook Messenger, and Slack [2]. They may use text or voice-assisted interfaces or may include an embodied agent using virtual characters to simulate both verbal and nonverbal aspects of human communication [4]. CAs can be further classified as simple rule-based agents or smart, artificial intelligence (AI)–based agents using natural language processing or machine learning to generate the responses [2].

Following the trends in other industries, health care has seen increasing adoption of CAs in recent years [1]. Health care CAs are versatile tools able to cater to several health needs, such as providing timely information [5], supporting mental health disorder management [6,7], assisting with triage in clinical settings [8,9], supporting chronic disease self-management, or delivering lifestyle change interventions, such as physical activity [10] and dietary changes, that increasingly incorporate elements of behavior change in the intervention design. In general, health care CAs appear to be effective in improving individuals’ outcomes [11,12] and are acceptable to users, who often describe them as friendly and trustworthy.

Increasingly, health care CAs are used to deliver behavior change interventions, defined as complex interventions, comprising an interplay of 1 or several heterogeneous behavior change techniques (BCTs) [13]. BCTs are “observable and replicable components designed to change behavior” [13]. BCTs are considered the smallest active ingredient in an intervention, and can be used alone or in combination with other BCTs [13]. Adequate categorization of the BCTs included in an intervention allows for more efficient coding, leading to easier replication when designing similar interventions [13]. Several methods to classify BCTs have been developed, of which the Behavior Change Technique Taxonomy version 1 (BCTTv1) [14] is the most established and commonly used.

Several reviews have synthesized the evidence about behavior change interventions delivered by digital health tools and CAs, such as a systematic review reporting on the use of BCTs in effective digital diabetes prevention interventions [15], a mapping review offering a description of the current uses of CAs for behavior change [16], and a scoping review describing the use of embodied CAs to support healthy lifestyle [17]. These reviews presented descriptive data, without an in-depth analysis of the type of BCTs used in the interventions, the use of behavior change theories to guide the interventions, the frequency with which each BCT was used, and potential associations between BCTs and intervention effectiveness. Therefore, this scoping review aims to analyze the use of BCTs in behavior change interventions delivered by CAs; specifically, it describes the health behaviors and disorders targeted by the intervention, describes the types of CAs used to deliver the behavior change interventions, identifies the theories or frameworks guiding the design of the behavior change interventions, identifies the most common type of BCTs used in CA-delivered interventions in health care, compares the BCTs employed in different types of CAs and for different health disorders, and compares the BCTs employed in the experimental and comparison interventions of studies evaluating CA-delivered behavior change interventions.

## Methods

### Overview

The scoping review was performed according to the Joanna Briggs Institute guidelines [18] and reported in alignment with the PRISMA-ScR (Preferred Reporting Items for Systematic Reviews and Meta-Analyses extension for Scoping Reviews) reporting guidelines (Multimedia Appendix 1) [19]. The protocol was registered in Open Science Framework Registries [20] in April 2021 and was published in a peer-reviewed journal in July 2021 [21].

### Search Strategy

The search strategy was designed using a comprehensive list of words and phrases that define CAs (Multimedia Appendix 2). We searched PubMed, Embase (Ovid), and CENTRAL (Cochrane Central Register of Controlled Trials), from their inception, and the first 10 pages of Google and Google Scholar [22,23] on April 26, 2021.

### Eligibility Criteria

This scoping review included primary, experimental studies in English evaluating the use of CAs to deliver health care interventions focusing on behavior change. Eligible study designs were randomized controlled trials (RCTs), quasi-RCTs, cluster-randomized trials, controlled before-and-after studies, uncontrolled before-and-after studies, interrupted time series, and pilot and feasibility studies. We excluded nonexperimental study designs, such as observational studies, qualitative studies, opinion pieces, editorials, conference abstracts, and secondary studies.

We included studies on text-based, voice-based, and embodied CAs, defined as conversational interfaces featuring a human-like avatar able to mimic the verbal and nonverbal components of a face-to-face conversation [24]. The eligible studies reported any health care intervention focused on behavior change to improve or promote a healthy lifestyle, or to support the management of physical or mental health conditions. Lastly, behavior change was an essential aspect of the eligible studies, with or without reference to an associated behavior change theory, in line with previous research in this area [25]. The BCTs were coded according to the BCTTv1 [14]. The taxonomy consists of 93 BCTs grouped into 16 distinct categories, aimed at providing a cross-domain template to facilitate research and intervention replication.

### Screening, Data Extraction, and Analysis

#### Screening

Screening for eligibility was performed in 2 stages. First, 2 researchers (NYWL and WWTG) worked independently to screen the titles and abstracts of all retrieved studies using Covidence [26]. Studies were excluded if their focus or study design did not align with our predefined eligibility criteria. Studies included in the first round of screening were uploaded to EndNote X9 (Clarivate), and the full-text papers were retrieved and screened for eligibility by 3 researchers working independently (AIJ, NYWL, and WWTG). Discrepancies in any screening stage were resolved through discussions between the reviewers, or by engaging a fourth reviewer (LM). The search and screening processes were documented in a study selection flowchart [27].

#### Data Extraction

The data were extracted using a Microsoft Excel (Microsoft Corporation) form developed by the research team, based on a data extraction form used in a previous scoping review [2], and a section on behavior change was added. The form was piloted in 3 studies and amended according to team members’ feedback before being used for data extraction. Reviewers worked in pairs (AIJ worked with LM and NYWL worked with WWTG) to extract data from 10 papers (20%) and individually for the remaining 42 papers (80%). Data extracted by all reviewers were subsequently reexamined by 2 researchers (LM and AIJ). Reviewers met regularly during this process to ensure a common understanding of the data extraction process and the concordance of the extracted data. The data extracted by each pair of reviewers were compared, and any disagreements were resolved through consensus or consultation with a third reviewer, acting as an arbiter.

The data extraction form contained the following items: first author, year of publication, title of the article, study design, target disorder, description of the behavior change intervention, CA name, delivery channel, dialog technique, input and output modalities, end goal of the intervention, use of behavior change theories or frameworks, and BCTs mapped according to the BCTTv1 [14].

#### Data Analysis

Data were analyzed using descriptive statistics and frequent itemset mining (FIM) to explore possible BCT clustering [28]. Data were presented in a diagrammatic or tabular form accompanied by a narrative summary.

#### Frequent Itemset Mining

The FIM analysis was performed by implementing the Apriori algorithm using the *arules* package version 1.7-1 [29] in R version 4.1.2 (R Foundation for Statistical Computing) [30]. FIM aims to find patterns or associations in a group of items (itemset) by sorting the items that frequently appear together in the data set. The analysis starts by calculating support (how frequently an item appears in the data set) and confidence (number of times individual items “x” and “y” appear together in the data set) thresholds and discarding any itemset with support or confidence values below the predetermined minimum threshold.

For this analysis, we assessed the 10 most frequently appearing patterns, for the overall data set and for each clinical domain. For the overall data set, the minimum threshold for algorithm support and confidence was set at 0.10 and 0.90, respectively, or itemset appearing in at least 10% of the data set (≥4 studies) and appearing together at least 90% of the time. For each clinical domain, the minimum thresholds were 0.20 for support and 0.90 for confidence to account for the fewer number of studies in each sub data set [31].

## Results

### Overview of Search Strategy

**Figure 1:**
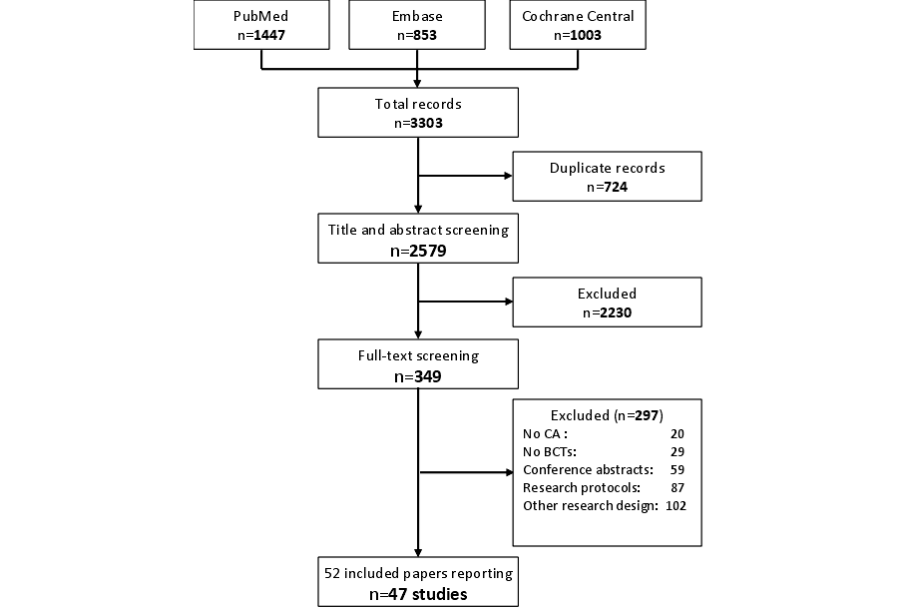
Study selection flowchart. BCT: behavior change technique; CA: conversational agent.

The search strategy retrieved 2579 papers after removing duplicates, of which 349 were eligible for full-text screening. Among these, 52 papers were finally included in this review. We reported 47 studies, as 4 studies were reported in 2 papers each and 1 study included a corrigendum. Figure 1 presents the study selection process.

### Characteristics of the Included Studies

Multimedia Appendix 3 presents a summary of the studies included in this review [6,11,32-79]. Over half of the studies (26/47, 55%) were published from 2019 onward [11,32,34,37,40,42-46,48-55,58-61,65,66,71,72,76-78], including 6 published in the first quarter of 2021 [42,46,49,54,55,60]. All papers except 1 [32] were published in high-income countries, and 24/47 studies (51%) were published in the United States [6,32,34,36,39,43,45,47, 48,51,52,54,56-58,61-64,67,69-75].

Most studies included a control group except 5/47 (11%) single-group pretest posttest trials [43,46,55,58,65,66], 3/47 (6%) feasibility studies [59-61], and 1/47 (2%) pilot study [48]. A total of 26/47 studies (55%) were RCTs [6,11,33,35-37,39-41,44,45,49,50,53,54,62-64,68-75,77,78]. In 36/47 studies (77%), the primary outcomes were associated with improvement of the target disorder [6,33,36, 38-45,47-59,62-64,67,68,70-75,77-80], 5/47 studies (11%) reported technical-related primary outcomes (eg, technical performance, system crashes) [11,60,65,66,69,76], and 6/47 studies (13%) reported primarily user experience outcomes (eg, engagement with the CA, user satisfaction) [32,34,35,37,46,61]. Most interventions aimed to support treatment or monitoring (22/47, 47%) [6,33,35-44,46,48-50,53-55,59,60,80] or to promote healthy lifestyle change (18/47, 38%) [11,32,34, 45,61-66,68-76,78,79]. Table 1 presents a summary of the included studies.

**Table 1:** Characteristics of included studies (N=47).

Study characteristics		Studies, n (%)
**Year of publication**		
	Before 2019	21 (45)
	2019 or after	26 (55)
**Country**		
	United States	24 (51)
	United Kingdom	6 (13)
	Japan	3 (6)
	Korea	3 (6)
	Switzerland	3 (6)
	Australia	2 (4)
	France	1 (2)
	Germany	1 (2)
	India	1 (2)
	Netherlands	1 (2)
	Spain	1 (2)
	Sweden	1 (2)
**Study design**		
	Randomized controlled trial	26 (55)
	Pilot study	9 (19)
	Single-group pretest posttest trial	5 (11)
	Feasibility study	5 (11)
	Microrandomized controlled trials	1 (2)
	Nonrandomized comparison study	1 (2)
**Study outcomes**		
	Clinical	23 (49)
	Clinical; user experience	12 (26)
	User experience; clinical	6 (13)
	Technical; clinical	3 (6)
	Technical; clinical; user experience	2 (4)
	Clinical; technical	1 (2)
**Clinical focus of the interventions**		
	Lifestyle behavior change	17 (36)
	Treatment and monitoring	16 (34)
	Treatment and monitoring + education	4 (9)
	Education	4 (9)
	Education + lifestyle behavior change	3 (6)
	Treatment and monitoring + lifestyle behavior change	2 (4)
	Education + treatment and monitoring	1 (2)
	Lifestyle behavior change + education	1 (2)
**Clinical domains**		
	Mental health	19 (40)
	Chronic disorders	14 (30)
	Lifestyle modification	14 (30)

### Clinical Domains

#### Mental Health Interventions

Most CAs focused on mental health (19/47, 40%) [6,32-47,79,80], either supporting mental well-being (5/19, 26%) for healthy individuals [46,47,79,80] or patients recovering from cancer [33]; enabling self-improvement interventions such as problem solving [34] or communications skills [35]; or assisting participants in the management of a mental health disorder (14/19, 74%) [6,36-46], including depression (with or without anxiety; 3/19, 16%) [6,36,37], emotional distress (2/19, 11%) [38,39], bipolar disorder [40], panic disorder [41], fear of heights [42], adult attention deficit disorder [43], substance use disorder [44], gambling [45], and social exclusion [46].

All except 2 interventions [44,47] included a control group, and 10/19 studies (53%) were RCTs [6,33-37,39,41,45,46]. A total of 6 studies included an active comparison with another digital intervention [34,38,39,46], a paper-based version of the CA intervention [40], or mood monitoring [33]. Besides, 6 studies provided information about the target disorder [6,35,37,41,43,48], and 10 experimental interventions (10/17, 59%) were reported as more effective than the comparisons [6,33-37,39,41,45,46].

#### Chronic Disorder Management Interventions

A total of 14/47 studies (30%) offered interventions focusing on a chronic disease other than mental illness [49-63]. Most studies (4/14, 29%) targeted a metabolic disorder including obesity (n=1) [63], prediabetes (n=1) [62], or type 2 diabetes (n=2) [51,56]. Three studies evaluated a pain management intervention for osteoarthritis (n=2) [57,58] or for general management of chronic pain (n=1) [54]. Other studies focused on asthma [61], atrial fibrillation [52,53], HIV [49], hypertension [50], insomnia [60], irritable bowel syndrome [55], and prostate cancer [59]. The interventions aimed to support treatment and monitoring tasks (8/14, 57%) or provide education (4/14, 29%).

Half of the included studies were feasibility or pilot studies, and 5/14 studies (36%) were RCTs [49,50,53,54,62]. Comparison interventions included a nurse-led instruction mirroring the CA intervention [50], physical activity monitoring using a pedometer [63], provision of information [57,58], treatment as usual [51-53], and waitlist controls [54,55]. Furthermore, 6/14 studies (43%) were single-group interventions without a comparison group [48,55,58-61]. Only 2 studies described the experimental interventions as more effective than the comparisons (2/8, 25%) [51,52,54].

#### Lifestyle Change Interventions

A total of 14/47 studies (30%) included interventions to support lifestyle modification [11,64-79], particularly increasing physical activity (10/14, 71%), either as the sole intervention (n=6) [64,69,74-77,79] or in combination with another approach such as diet improvement (n=2) [65-67], or diet improvement plus stress relief (n=1) [70]. Four studies (4/14, 29%) targeted an aspect of women’s health including preconception care (n=3) [71-73,78] and breastfeeding support (n=1) [68]. One study offered a smoking cessation intervention [11]. In 12 studies, the interventions aimed to facilitate lifestyle change (12/14, 86%) [11,63-76,78], while 2 studies offered education [67,77].

Among this, 1/14 (7%) study was a single-group pretest-posttest trial [65,66], while most studies (11/14, 79%) were RCTs [11,63,64,68-75,77,78]. In 7/13 studies (54%) comparison interventions consisted of face-to-face versions of the intervention [74-76], abridged interventions that excluded the CA [11,64,65,70], or a similar version of the intervention with differing reward systems [77,79]. Other comparisons included information-only interventions (3/13, 23%), treatment as usual (1/13, 8%), or waitlists (2/13, 15%). Most experimental interventions were reported to be more effective than the comparisons (9/13, 69%).

### Characteristics of CAs

Table 2 summarizes the characteristics of the included CAs.

A total of 39 CAs were included. Six CAs were reported in 2 or more manuscripts. Four CAs (Carmen [74-76], Tanya [52,53,68], Tess [37,62], and Todaki [41,43]) were reported in 2 papers each, and 2 CAs (Gabby [70-73] and MYLO [34,38,39]) were reported in 3 manuscripts. Three CAs were adapted for different target disorders. Embodied CA Tanya was used as an educational tool for patients with atrial fibrillation [52,53] and to offer breastfeeding support [68], CA Tess was used for mental health [37] and diabetes care [62], and Todaki was used to deliver CBT for panic disorder [41] and to manage adults with attention deficit disorder [43]. Finally, MYLO was used in student and older adult [38] populations by 2 distinct research groups.

The majority of CAs featured 1 or more anthropomorphic characteristics, such as the assignation of gender, name, or a human-like display. Most CAs (41/47, 87%) responded to a name, 27/47 CAs (57%) were presented as female agents, and 20/47 (43%) were embodied CAs. Most CAs used rule-based algorithms to design the flow of conversations, either by themselves (35/47, 75%) or complemented with AI (2/47, 4%). CAs were more often available through a smartphone app (14/47, 30%) or web page (13/47, 28%). In all but 3 CAs (44/47, 94%), the primary method for users’ inputs was text; 7/47 of these CAs (15%) also accepted verbal or visual inputs, whereas 3/47 CAs (6%) received only verbal inputs. Almost 80% of all CAs (36/47, 77%) displayed a “coach-like” personality, characterized by an encouraging, motivating, and nurturing conversational style.

**Table 2:** Characteristics of CAs^a^ (N=47).

CA characteristics		Values, n (%)
**Type of CA**		
	Embodied CAs	20 (43)
	No visual representation	12 (26)
	Human-like cartoon avatar	10 (21)
	Nonhuman cartoon avatar	5 (11)
**Gender**		
	Female	27 (57)
	No gender assigned (no avatar/no human avatar)	16 (34)
	Male	2 (4)
	Defined by the user	2 (4)
**CA “level of intelligence”**		
	Rule-based CAs	34 (72)
	Artificial intelligence CAs	9 (19)
	Rule-based + artificial intelligence CAs	4 (9)
**Dialog modality**		
	Predetermined text	28 (60)
	Free text	8 (17)
	Predetermined and free text	7 (15)
	Not specified	4 (9)
**Delivery channel**		
	Smartphone app	14 (30)
	Web based	13 (28)
	Desktop	7 (15)
	Messaging apps	6 (13)
	Two or more delivery channels	6 (13)
	Tablet computer	1 (2)
**Users’ input modalities**		
	Text	37 (79)
	Text + others (voice, images, video)	7 (15)
	Voice (± video)	3 (6)
**CA output modalities**		
	Text + others (voice, images, video)	29 (62)
	Text	15 (32)
	Voice (± images, video)	3 (6)
**CA personality**		
	Coach like	36 (77)
	Health care professional like	9 (19)
	Not specified	2 (4)

^a^CA: conversational agent.

### Type of CA and Clinical Domains

Embodied CAs were used to deliver almost two-thirds (9/14, 64%) of the interventions promoting lifestyle modification [64,65,68-76], 43% (6/14) of the chronic disease management interventions [49,51-53,59,60,63] and only 26% (5/19) of the mental health interventions.

By contrast, most mental health CAs did not include an avatar (8/19, 42%) [34,35,38-40,45,47,81], or they were represented by a nonhuman avatar (5/19, 26%) [6,33,41,43,44]. Human-like avatars were present in 1/19 (5%) mental health intervention [37], 6/14 (43%) chronic disease management interventions [54,55,57,58,61,62], and 3/14 (21%) lifestyle change interventions [66,67,77,78].

### Behavior Change Theories and Techniques

#### Behavior Change Theories

A total of 12/47 (26%) studies incorporated a behavior change theory to guide the CA intervention design, including 4/14 (29%) studies targeting a chronic disorder [51,54,59,61], 7/14 (50%) studies [65,71-76,78,79] evaluating a lifestyle change intervention, and 1/19 study (5%) [37] on mental health. The Transtheoretical Model was the most used behavior change theory, either alone [37,71-73,78] or together with the Social Cognitive Theory [51,65,74-76]. In addition, 4/19 (21%) mental health studies and 2/14 (14%) studies targeting a chronic disorder based their interventions on theories derived from the behavior [34,38,39], communication [57,58], learning [59], or psychological domains [33] (Table 3).

The use of theories aimed to guide the design of the intervention or to monitor participants’ stages of change as they progressed through the intervention, as exemplified by 3 studies [71-73,78] using the Transtheoretical Model and 1 study using the Health Action Process Approach [54]. It was not clear how the use of theories influenced the intervention design or the choice of BCTs. For example, 4 studies using the Transtheoretical Model included a wide variety of BCTs, ranging from 3 [78] to 10 [72,73]. Similarly, 4 studies [51,65,74-76] using the Transtheoretical Model and the Social Cognitive Theory incorporated between 6 [51] and 19 [75,76] BCTs.

**Table 3:** Behavior change theories informing the CA^a^-based interventions (N=47).

Theories guiding CA interventions		Studies, n (%)
No theory		29 (62)
**Behavior change theories**		11 (23)
	Transtheoretical Model	4 (9)
	Transtheoretical Model + Social Cognitive Theory	4 (9)
	Theory of Planned Behavior + Self-Determination Theory + Technology	1 (2)
	Acceptance theories	
	Health Action Process Approach	1 (2)
	Habit Formation Model	1 (2)
**Behavior change theories + other theories**		1 (2)
	Unified Theory of Acceptance and Use of Technology + Cognitive Theory	1 (2)
	Multimedia Learning	
**Other theories**		6 (13)
	Perceptual Control Theory	3 (6)
	Communication Accommodation Theory	2 (4)
	Stress and Coping Theory + Broaden and Build Theory of Positive Emotion	1 (2)

^a^CA: conversational agent.

#### Incorporated BCTs

**Figure 2:**
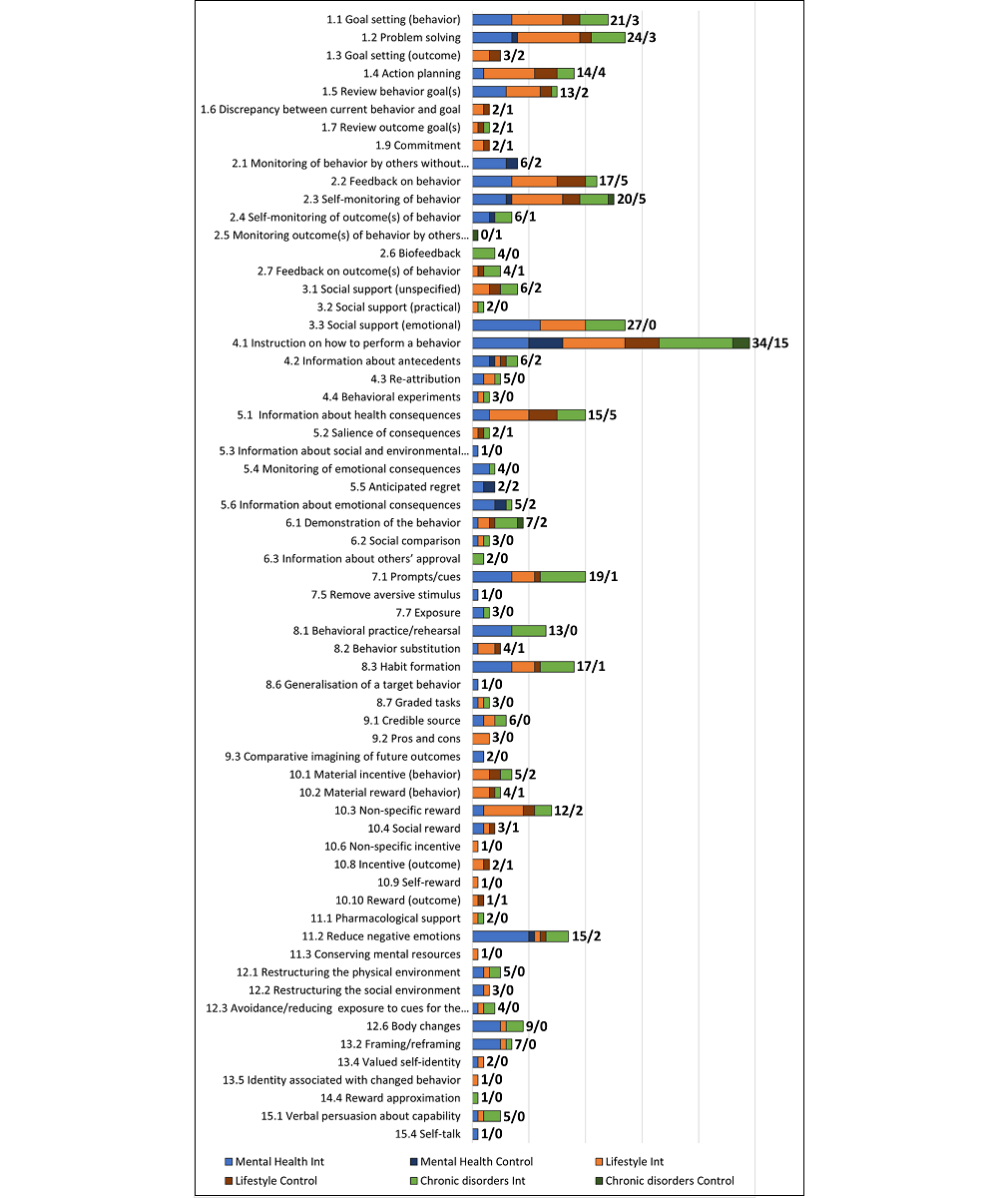
Number of studies using each BCT in the experimental and comparison interventions. BCT: behavior change technique; Int: intervention.

The experimental interventions incorporated 63 BCTs from 15 categories, whereas the comparison interventions included 32 BCTs from 10 categories. However, only 24 BCTs were incorporated into experimental interventions in 5 or more studies, whereas 12 BCTs were reported in only 1 study each. The most incorporated BCT across interventions was 4.1 “Instruction on how to perform a behavior” (34/47, 72%), followed by 3.3 “Social support (emotional)” (27/47, 57%) and 1.2 “Problem solving” (24/47, 51%), whereas only 1 study included a BCT from category 14 (14.4 “Reward approximation”) in the experimental intervention, and none included BCTs from category 16 “Covert learning.” Figure 2 shows the frequency of presentation of all 63 BCTs in experimental and comparison interventions.

The average number of BCTs included in the experimental interventions was 9 (range 2-21 BCTs). By contrast, comparison interventions (n=38) included an average of 2 BCTs (range 0-17 BCTs).

#### Use of BCTs According to the Clinical Domain

The number of BCTs in experimental interventions was consistent across all clinical domains. Mental health interventions included an average of 8 BCTs (range 3-16 BCTs), chronic disorder management interventions included an average of 9 BCTs (range 2-18 BCTs), and lifestyle change interventions included an average of 10 BCTs (range 3-21 BCTs). The number of BCTs included in comparison interventions varied from an average of 2 BCTs in chronic disorder management (range 1-3 BCTs) and mental health interventions (range 1-2 BCTs) to a mean of 6 BCTs (range 1-17 BCTs) in lifestyle change interventions.

Mental health interventions incorporated 41 BCTs in experimental interventions. The most common BCTs were 3.3 “Social support (emotional)” (12/19, 63%), 11.2 “Reduce negative emotions” (11/19, 58%), 4.1 “Instruction on how to perform a behavior” (9/19, 47%), and BCTs 1.1 “Goal setting (behavior),” 1.2 “Problem solving,” 2.2 “Feedback on behavior,” 7.1 “Prompts/cues,” 8.1 “Behavioral practice/rehearsal,” and 8.3 “Habit formation” that were included in 7/19 (37%) studies each.

Lifestyle change interventions included 46 BCTs. The most common BCT was 1.2 “Problem solving” (11/14, 79%), followed by 4.1 “Instruction on how to perform a behavior” (10/14, 71%) and BCTs 1.1 “Goal setting (behavior),” 1.4 “Action planning,” and 2.3 “Self-monitoring of behavior,” included in 9/14 (64%) studies each.

Chronic disorder management interventions included a total of 41 BCTs. Almost all studies included BCT 4.1 “Instruction on how to perform a behavior” (13/14, 93%), followed by 7.1 “Prompts/cues” (8/14, 57%), 3.3 “Social support (emotional)” (7/14, 50%), and BCTs 1.2 “Problem solving,” 8.1 “Behavioral practice/rehearsal,” and 8.3 “Habit formation,” all included in 6/14 studies (43%).

**Figure 3:**
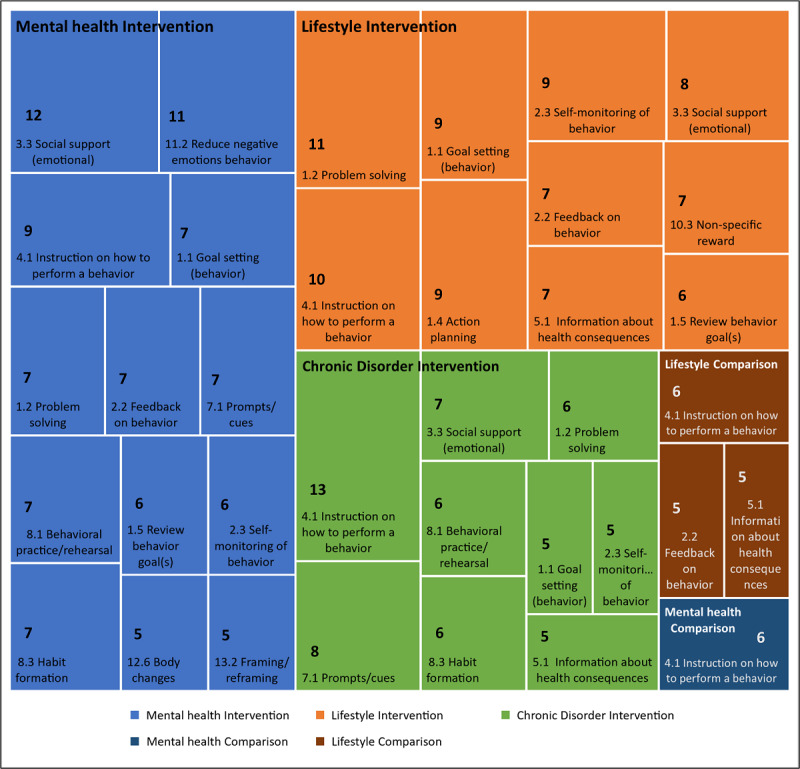
Commonly used BCTs according to the clinical domain. BCT: behavior change technique.

Figure 3 presents a summary of the most commonly used BCTs according to the clinical domain. Multimedia Appendix 4 presents a table summarizing the use of each BCT according to the clinical domain.

#### BCT Clustering According to the Clinical Domain Using FIM

The overall data set (n=47) generated 206 rules with an average support of 0.12, suggesting that the rules applied to at least 12% of the data set or about 6 studies. In general, 26% of the studies included BCTs 4.1 “Instruction on how to perform a behavior” and 8.1 “Behavioral practice/rehearsal,” whereas 23% of the studies included BCTs 4.1 “Instruction on how to perform a behavior,” 7.1 “Prompts/cues,” and 8.3 “Habit formation.”

The mental health domain (n=19) generated 45 rules with an average support of 0.22. About one-quarter of studies (26%) included 1 of 3 rules: the first itemset included BCTs 1.5 “Review behavior goal(s),” 2.2 “Feedback on behavior,” and 3.3 “Social support”; followed by the itemset comprising BCTs 3.3 “Social support” and 12.6 “Body changes”; and the itemset containing BCTs 3.3 “Social support,” 4.1 “Instruction on how to perform a behavior,” and 11.2 “Reduce negative emotions.” Conversely, the lifestyle change domain (n=14) generated 1322 rules with an average support of 0.24. About 64% of the studies included BCTs 1.2 “Problem solving” and 2.3 “Self-monitoring of behavior,” whereas 57% of the studies also included BCT 1.1 “Goal-setting (behavior).” Finally, the chronic disorder management domain (n=14) generated 230 rules with an average support of 0.23. Most studies (93%) included BCT 4.1 “Instruction on how to perform a behavior,” whereas 57% also included BCT 7.1 “Prompts/cues.”

Multimedia Appendix 5 presents a table describing the top 10 itemsets for all included papers and each clinical domain.

#### Use of BCTs According to the CA Type

Interventions delivered by any type of CA included an average of 9 BCTs. However, the number of BCTs in experimental interventions varied by type of CA: embodied CAs included 2-19 BCTs, CAs represented by an avatar included 3-14 BCTs, and CAs with nonspecified or nonvisual representation incorporated 4-21 BCTs.

Embodied CAs included a total of 49 BCTs in the interventions. The most common BCTs were 3.3 “Social support (emotional) (14/20, 70%), and BCTs 1.2 “Problem solving,” 2.3 “Self-monitoring of behavior,” and 4.1 “Instruction on how to perform a behavior,” which were found in 13/20 (65%) studies each. By contrast, CAs represented by an avatar included a total of 38 BCTs in the interventions. The most common BCTs were 4.1 “Instruction on how to perform a behavior” (13/15, 87%), and BCTs 3.3 “Social support (emotional)” and 7.1 ”Prompts/cues” included in 10/15 (67%) studies each. Finally, CAs with nonspecified or nonvisual representation incorporated a total of 47 BCTs. Four BCTs (1.2 “Problem solving,” 4.1 “Instruction on how to perform a behavior,” 7.1 ”Prompts/cues,” and 8.3 “Habit formation”) were included in 6/12 (50%) studies, and BCT 11.2 “Reduce negative emotions” was included in 5/12 (42%) studies. Multimedia Appendix 6 provides further information about the use of BCTs according to the type of CA.

## Discussion

### Principal Findings

This scoping review included 47 studies reporting behavior change interventions delivered by CAs, targeting chronic disorders, lifestyle change, and mental health. The interventions included a total of 63 BCTs, but only 24 were consistently found in 5 or more interventions. The BCTs represented aspects of health education (BCT 4.1), self-management (BCTs 1.1, 1.2, and 2.3), and social support (BCT 3.3). Several behavior change theories informed the intervention design in 12/47 (26%) studies of the included studies. However, studies informed by the same theory employed different sets of BCTs. Our findings align with previous systematic reviews reporting that similar BCTs were frequently incorporated into effective lifestyle change interventions [82], or into digitally delivered interventions [15].

We did not find a relationship between the use of theories, the type of theory used, and the number and type of BCTs included in the interventions. Furthermore, a small number of studies [11,61] guided the intervention design, using modified BCT taxonomies that addressed smoking cessation [11] and diet modification [61]. These data suggest that the choice of BCTs may be primarily determined by the target behavior rather than the use of a behavior change theory. The impact of using a behavior change theory is nevertheless unclear. A 2010 systematic review [83] reported that the use of a behavior change theory was associated with increased effectiveness of the interventions, although just over 20% of studies included a theory. Conversely, a systematic review by Van Rhoon et al [15] reported the use of theories in 16/21 (76%) studies but did not assess intervention effectiveness. In addition, a recent overview of systematic reviews [84] reported the use of theories in the intervention design of 19%-52% of the included studies, although there was no clear association with the intervention effectiveness.

The categorization of studies in 3 distinct clinical domains suggested different prioritizations in mental health, lifestyle change, and chronic disorders, although the delivery of health education, evidenced by the frequent occurrence of BCTs 4.1 “Instruction on how to perform a behavior,” 8.1 “Behavioral practice/rehearsal,” and 8.3 “Habit formation,” was consistent across all clinical domains.

Mental health interventions frequently included BCTs 3.3 “Social support (emotional)” and 11.2 “Reduce negative emotions.” Specifically, BCT 3.3 may be associated with the use of psychotherapeutic techniques such as cognitive behavioral therapy or motivational interviewing, while the inclusion of BCT 11.2 suggests the use of relaxation techniques and mindfulness to support stress management and emotional regulation. Therefore, behavior change in mental health settings appeared to be closely interlinked with the therapeutic strategies. Concurrently, the inclusion of other BCTs, such as instructions to perform a behavior (BCT 4.1), goal setting (BCT 1.1) and reviews (BCT 1.5), problem solving (BCT 1.2), and feedback (BCT 2.2), may be aligned with general principles of patient participation in decision making [85], as well as highlight the importance of health education [86,87], particularly relevant in self-initiated digital interventions.

Lifestyle change interventions frequently included problem-solving (BCT 1.2) techniques to help users better understand their barriers to behavior change, and goal setting (BCT 1.1) and self-monitoring (BCT 2.3) to work toward the target behavior. These BCTs were often included together and this may suggest a synergistic relationship. At the same time, the importance of ensuring adequate health literacy to improve population outcomes was emphasized by the frequent inclusion of BCT 4.1 “Instruction on how to perform a behavior.”

Chronic disorder management interventions favored not only the inclusion of instructional BCTs, such as guidance to perform a target behavior (BCT 4.1) but also reminders (BCT 7.1 “Prompts/cues”) to facilitate the acquisition of new routines (BCT 8.3 “Behavioral practice/rehearsal”). Self-management of chronic illnesses is essential to ensure improved patient outcomes and adequate quality of life but requires that individuals engage in a steep learning curve as they adapt to living with a long-term condition and develop new habits.

In general, the relationship between the number and type of BCTs and the effectiveness of the interventions was inconsistent and appeared to be determined by the clinical domain. Effective lifestyle change interventions tended to include a higher number of BCTs, a finding that was not replicated in the other clinical domains. At the same time, lifestyle change interventions were comparatively more effective than those in other clinical domains, particularly chronic disorders. Effective interventions in the lifestyle change and mental health domains frequently included BCTs related to goal setting and planning, timely provision of feedback, health education, and rewards on completed tasks. Previous studies reported varied results. A 2017 systematic review of 48 studies [82] evaluating the management of overweight and obesity in adults found small pooled effect sizes for short- and long-term diet and physical activity interventions. Effective interventions included a larger number of BCTs, particularly BCTs encouraging goal setting and self-monitoring of behavior. Similarly, a systematic review on the BCTs and technical features of digital interventions for the prevention of type 2 diabetes [15] found that effective interventions included a larger number of BCTs or BCTs related to social support, goal setting, and feedback.

There was an unexpected relationship between the CA types and the clinical domain, manifested by a predominance of embodied CAs in lifestyle change interventions, and the use of nonhuman or nonavatar CAs in mental health interventions. The reasons for these findings are unclear and beyond the scope of this review; however, further research may help clarify the role of avatars, or virtual humans, if any, in delivering behavior change interventions. Other reviews have reported the use of embodied CAs to support mental health interventions, particularly autism [20,24], but methodological differences limit the comparisons with our findings. Provoost et al’s scoping review [4] used a broader definition of embodied CA, while a systematic review by Laranjo et al [87] included only AI-based CAs.

### Strengths and Limitations

This scoping review has several strengths. First, we used a comprehensive literature search of peer-reviewed and gray literature that prioritized the sensitivity of the search terms to capture a broad range of publications reporting the use of CAs in health care. However, relevant studies may have been omitted. Second, we included studies reporting on a wide variety of physical and mental health conditions, and categorized the studies into 3 distinct clinical domains, revealing differences in the type of BCTs selected in each domain.

There are also some limitations. First, many studies did not provide exact BCT codes when describing the interventions, therefore categorization of BCTs was inferred from the paper’s description by the research team, based on thorough analysis, rigorous team discussion, and reviews to establish consensus. Second, given the descriptive nature of scoping reviews, we were unable to explore in more depth the relationship between the choice of BCTs and the effectiveness of the intervention, or the type of CA used to deliver the intervention.

### Future Research and Practice Recommendations

This review has highlighted several areas that warrant further research. First, reporting guidelines to ensure accurate reporting of the BCTs included in behavior change interventions according to standardized taxonomies, such as the BCTTv1 [14], should be implemented. Such guidelines would facilitate reproducibility of research, assessment of active intervention components, and evidence synthesis. Second, further research is needed to increase our understanding of the impact of behavior change theories in the design of interventions, the choice of BCTs, and the effectiveness of the intervention. Third, the impact of CAs to deliver behavior change interventions should be further explored, particularly the influence of a conversational interface on engagement, adherence, and effectiveness of the intervention when compared with less interactive digital technologies. Furthermore, comparisons between rule-based CAs and those incorporating machine learning or natural language processing should be further investigated. Fourth, the possible role of the type of CA in delivering behavior change interventions, as suggested in our findings, should be further explored. Fifth, the relationship between the ideal combination of BCTs required to design effective interventions may be evaluated using data mining techniques such as FIM or multiple correspondent analysis. Lastly, the relationship between behavior change interventions and mental health requires further evaluation.

The use of CAs to deliver behavior change interventions appears promising, particularly to support lifestyle change, although better reporting of BCTs included in the interventions is warranted to facilitate analysis of active components, design more effective interventions, and ensure reproducibility of research. The role of CA types in delivering behavior change interventions should be further explored.

## Appendix

Multimedia Appendix 1 PRISMA-ScR (Preferred Reporting Items for Systematic Reviews and Meta-Analyses extension for Scoping Reviews) checklist.

Multimedia Appendix 2 PubMed search strategy.

Multimedia Appendix 3 Characteristics of included studies.

Multimedia Appendix 4 Use of BCTs according to the clinical domain. BCT: behavior change technique.

Multimedia Appendix 5 Frequent Itemset Mining (FIM).

Multimedia Appendix 6 Use of BCTs according to the CA type. BCT: behavior change technique; CA: conversational agent.

## Abbreviations

AI: artificial intelligence
BCT: behavior change technique
BCTTv1: Behavior Change Technique Taxonomy version 1
CA: conversational agent
CENTRAL: Cochrane Central Register of Controlled Trials
FIM: frequent itemset mining
PRISMA-ScR: Preferred Reporting Items for Systematic Reviews and Meta-Analyses extension for Scoping Reviews
RCT: randomized controlled trial
