# Supplementary material for: Conversational Agents in Health Care: Scoping Review of Their Behavior Change Techniques and Underpinning Theory
Source: J Med Internet Res. 2022 Oct 3;24(10):e39243. doi: 10.2196/39243 (PMC9577715; doi:10.2196/39243)
Supplement: Multimedia Appendix 2 [file jmir_v24i10e39243_app2.docx]

# Multimedia Appendix 2

## PubMed search strategy

1. Artificially intelligent chatbot[Title/Abstract] OR Artificially intelligent chat agent[Title/Abstract] OR Automated virtual agent[Title/Abstract] OR Automated chat agent[Title/Abstract] OR Artificial intelligence chatbot[Title/Abstract] OR Artificial conversational entity[Title/Abstract] OR AI agent[Title/Abstract]

2. Chatterbox[Title/Abstract] OR Chatbot[Title/Abstract] OR Chatterbot[Title/Abstract] OR Chat assistant[Title/Abstract] OR chat bot[Title/Abstract] OR chat-bot[Title/Abstract] OR chatter bot[Title/Abstract]

3. Conversational agent[Title/Abstract] OR Conversational system[Title/Abstract] OR Conversational assistant[Title/Abstract] OR Conversational User Interface[Title/Abstract] OR Conversational Personal Assistant[Title/Abstract] OR Conversational interface[Title/Abstract] OR Conversational avatar[Title/Abstract] OR Conversational computer[Title/Abstract] OR Conversational humanoid[Title/Abstract] OR Conversational Character[Title/Abstract] OR Conversational bot[Title/Abstract] OR Conversational AI[Title/Abstract]

4. Dialog system[Title/Abstract] OR Dialogue system[Title/Abstract]

5. Embodied agent[Title/Abstract] OR Embodied conversational agent[Title/Abstract]

6. Interactive online character[Title/Abstract] OR Interactive virtual agent[Title/Abstract] OR Interactive agent[Title/Abstract] OR Interactive conversational assistant[Title/Abstract]

7. Intelligent virtual agent[Title/Abstract] OR Intelligent virtual assistant[Title/Abstract] OR Intelligent conversational assistant[Title/Abstract] OR Intelligent conversational avatar[Title/Abstract] OR Intelligent agent[Title/Abstract] OR Intelligent assistant[Title/Abstract]

8. Smartbot[Title/Abstract] OR Smart bot[Title/Abstract] OR Smart virtual assistant[Title/Abstract]

9. Online chat agent[Title/Abstract] OR Sociable agent[Title/Abstract] OR Relational agent[Title/Abstract]

10. Talk bot[Title/Abstract] OR Talking avatar[Title/Abstract]

11. Text-based healthcare chatbot[Title/Abstract] OR Text based dialogue system[Title/Abstract] OR Text-based synchronous chat[Title/Abstract] OR Speech recognition software[Title/Abstract] OR Voice recognition software[Title/Abstract]

12. Virtual assistant[Title/Abstract] OR Virtual agent[Title/Abstract] OR Virtual advisor[Title/Abstract] OR Virtual conversational agent[Title/Abstract] OR Virtual coach[Title/Abstract] OR Virtual online assistant[Title/Abstract] OR Virtual personal assistant[Title/Abstract]

13. #1 OR #2 OR #3 OR #4 OR #5 OR #6 OR #7 OR #8 OR #9 OR #10 OR #11 OR #12
