# Supplementary material for: Conversational Agents in Health Care: Scoping Review of Their Behavior Change Techniques and Underpinning Theory
Source: J Med Internet Res. 2022 Oct 3;24(10):e39243. doi: 10.2196/39243 (PMC9577715; doi:10.2196/39243)
Supplement: Multimedia Appendix 3 [file jmir_v24i10e39243_app3.docx]

**Multimedia Appendix 3**: Characteristics of included studies

| Study ID [Ref] Country | Study design | Type of CA | CA name | CA Personality | Delivery channel | Target disorder | Goal of the  intervention | Behavior change theories | BCTs in intervention group | BCTs in comparison group | Intervention effectiveness |
| --- | --- | --- | --- | --- | --- | --- | --- | --- | --- | --- | --- |
| Ali 2020  USA  [33] | Pilot study | Not specified | Aging and Engaging Program (AEP) | Coach-like | Web based | Mental health | Improve social communication | Not mentioned | 2.2, 4.1, 8.1, 8.2 | 4.1 | Effective |
| Bendig 2021  Germany  [45] | Single group pretest-posttest trial | Not specified | Self-help to Uplift psychological wellbeing (“SISU”) | HCP-like | Wire | Mental health | Provide therapeutic writing intervention to improve mental wellbeing | Not mentioned | 2.1, 4.1, 5.4, 7.1, 8.3, 10.4, 13.2, 15.4 | NA | NA |
| Bennion 2020  UK  [36] | RCT | No avatar | MYLO | HCP-like | Web based | Mental health | Provide intervention based on the method of levels (MOL) therapy to improve emotional distress | Perceptual Control Theory | 1.2, 2.4, 4.3, 5.5, 5.6, 11.2 | 2.3, 5.5, 5.6 | No difference |
| Bickmore 2013-1  USA  [63] | RCT | ECA | Karen | Coach-like | Web based/ Desktop computer | Lifestyle | Deliver intervention to promote physical exercise and healthy diet | TTM, SCT | 1.1, 1.2, 1.4, 1.5, 2.3, 3.3, 4.1, 4.4 | 2.3 | Mixed |
| Bickmore 2013-2  USA  [62] | RCT | ECA | Steps to health | Coach-like | Tablet | Lifestyle | Deliver intervention to promote physical exercise | Not mentioned | 1.1, 1.2, 1.5, 1.6, 2.2, 2.3, 3.3, 8.3, 10.3 | 1.1 | Mixed |
| Bird 2018  Scotland  [32] | RCT | No avatar | MYLO | Coach-like | Web based | Mental health | Problem solving intervention to reduce problem-related distress | Perceptual Control Theory | 1.2, 2.4, 9.3, 11.2 | 1.2, 11.2 | No difference |
| Burton 2016  Scotland  [34] | RCT | ECA | Help4Mood | HCP-like | Desktop computer | Mental health | Supports depression management in-between appointments with healthcare provider | Not mentioned | 1.1, 1.4, 1.5, 2.2, 2.3, 3.3, 11.2 | None | Effective |
| Davis 2020 - Maher 2020  Australia  [64,65] | Single group pretest-posttest trial | Avatar | Paola | Coach-like | Slack app in smartphones | Lifestyle | Personalized physical activity and dietary coaching | Not mentioned | 1.1,1.2,1.4, 2.2, 2.3, 3.1, 4.1, 4.3, 7.1, 8.2, 9.1 | NA | NA |
| de Gennaro 2019  USA  [44] | RCT | ECA | Rose | Coach-like | Web based | Mental health | Provide social support after social exclusion | Not mentioned | 2.1, 3.3, 5.4 | 2.1 | Effective |
| Depp 2015  USA  [38] | RCT | No avatar | PRISM | Not mentioned | Smartphone app | Mental health | Early warning system to track mood fluctuations in users with bipolar disorders. | Not mentioned | 1.1, 1.5, 2.1, 7.1, 8.3 | 2.4 | Mixed |
| Dworkin 2019  USA  [47] | Pilot study | ECA | My Personal Health Guide | Coach-like | Smartphone app | Chronic disorders | Improve ART adherence in MSM | Not mentioned | 2.2, 2.3, 2.4, 2.6, 2.7, 3.2, 3.3, 4.1, 5.1, 7.1, 8.3, 9.1, 10.1, 11.2, 13.2, 15.1 | NA | NA |
| Echeazarra 2021  Spain  [48] | RCT | No avatar | TensioBot | Coach-like | Telegram | Chronic disorders | Blood pressure monitoring/ Instruction on adequate technique | Not mentioned | 2.3, 2.6, 4.1, 6.1, 7.1, 8.3, 15.1 | 2.5, 4.1, 6.1 | Mixed |
| Edwards 2013  USA  [66] | Pilot study | ECA | Tanya | Coach-like | Tablet/ Desktop computer | Lifestyle | Breastfeeding information and prenatal support in hospital | Not mentioned | 3.2, 3.3, 4.1, 6.1, 9.1, 10.6, 13.2 | 3.1, 4.1 | Effective |
| Fitzpatrick 2017  USA  [6] | RCT | Avatar | Woebot | Coach-like | Smartphone app/ Desktop computer | Mental health | Self-management of anxiety and depression for college students | Not mentioned | 1.1, 1.5, 2.2 3.3, 7.1, 8.3 10.3, 10.4, 11.2 | 4.1 | Effective |
| Freeman 2018  UK  [40] | RCT | ECA | Not specified | Coach-like | Desktop computer | Mental health | Guide users through exposure therapy for fear of heights | Not mentioned | 1.2, 2.3, 4.1, 4.2, 4.3, 4.4, 7.7, 8.1, 8.6, 8.7 | None | Effective |
| Friederichs 2014  Netherlands  [67] | RCT | Avatar vs no avatar | Not specified | HCP-like | Web based | Lifestyle | Improvement of physical activity using motivational Interviewing | Not mentioned | 1.2, 1.3, 1.4, 3.3, 4.3, 9.2, 12.2, 13.4, 15.1 | None | Effective |
| Fulmer 2018  USA  [35] | RCT | Avatar | Tess | Coach-like | Facebook Messenger/ Slack/ SMS | Mental health | Alleviate depression and anxiety symptoms using CBT and other therapies | TTM | 3.3, 4.1, 11.2, 12.6 | 4.1, 4.2 | Effective |
| Gaffney 2013  UK  [37] | Pilot study | No avatar | MYLO | HCP-like | Web based | Mental health | Problem solving intervention based on the principles of method of levels therapy to reduce problem-related distress | Perceptual Control Theory | 1.2, 2.4, 9.3, 11.2 | 5.5, 5.6 | Mixed |
| Gardiner 2017  USA  [68] | RCT | ECA | Gabby | Coach-like | Web based | Lifestyle | Deliver culturally aware preconception care advice | Not mentioned | 1.1, 1.2, 2.3, 3.3, 4.1, 4.2, 5.1, 11.2, 12.6 | 4.1, 4.2, 5.1 | Mixed |
| Gong 2020  Australia  [49] | RCT | ECA | Laura | Coach-like | Smartphone app | Chronic disorders | Diabetes self-management | TTM, SCT | 1.2, 2.2, 2.6, 2.7, 4.1, 5.1 | None | Mixed |
| Greer 2019  USA  [31] | Feasibility study | Avatar | Vivibot | Coach-like | Facebook Messenger | Mental health | Improve mental wellbeing in young people with cancer | Stress and Coping theory, BBTPE | 1.1, 3.3, 4.1, 7.1, 8.1, 11.2, 13.2 | 2.3 | Mixed |
| Guhl 2020 - Magnani 2020  USA  [50,51] | Pilot study | ECA | Tanya | Coach-like | Smartphone app | Chronic disorders | Health education, monitoring, and problem-solving for users with atrial fibrillation | Not mentioned | 1.1, 1.2, 4.1, 5.1, 7.1, 8.3 | None | Effective |
| Hauser-Ulrich 2020  Switzerland  [52] | RCT | Avatar | painSELfMAnagement (SELMA) | Coach-like | Smartphone app | Chronic disorders | Self-management of chronic pain | HAPA | 1.1, 2.3, 3.3, 4.1, 4.2, 5.2, 5.4, 6.3, 7.1, 8.1, 8.3, 12.6 | None | No difference |
| Hudlicka 2013  USA  [46] | Pilot study | ECA | Chris | Coach-like | Web based | Mental health | Mindfulness training and support to develop a practice routine | Not mentioned | 1.1, 1.2, 1.5, 2.2, 2.3, 3.3, 4.1, 5.1, 5.6, 6.1, 8.1, 8.3, 10.3, 11.2, 12.6, 15.1 | 4.1 | Effective |
| Hunt 2021  USA  [53] | Crossover RCT | Avatar | Zemedy | HCP-like | Smartphone app | Chronic disorders | Psychoeducation for patients with IBS. | Not mentioned | 1.2, 1.4, 3.3, 4.1, 4.2, 4.3, 4.4, 5.6, 6.1, 7.7, 8.1, 10.1, 11.2, 12.6 | None | Effective |
| Jack 2015  USA  [69] | RCT | ECA | Gabby | Coach-like | Web based | Lifestyle | Promotes behavior change for healthy preconception care | TTM | 1.1, 1.2, 1.4, 1.5, 2.3, 3.3, 4.1, 5.1 | 2.2 | Effective |
| Jack 2020 - Gardiner 2020  USA  [70,71] | RCT | ECA | Gabby | Coach-like | Web based | Lifestyle | Promotes behavior change for healthy preconception care | TTM | 1.1, 1.2, 1.4, 1.5, 2.2, 2.3, 3.3, 4.1, 5.1, 10.3 | 2.2 | Effective |
| Jang 2021  Korea  [41] | Feasibility study | Avatar | Todaki | Coach-like | Web based | Mental health | Psychoeducation and self-help skills for people with attention deficit | Not mentioned | 1.2, 1.4, 2.3, 3.3, 4.1, 4.2, 5.3, 7.1, 8.1, 8.3, 9.1, 11.2, 12.1, 12.6 | 4.1 | Effective |
| King 2013  USA  [72] | RCT | ECA | Carmen | Coach-like | Desktop computer | Lifestyle | Culturally aware physical activity advice and support for low-income older adults | TTM, SCT | 1.1, 1.2, 1.9, 2.2, 2.3, 5.1, 8.2, 10.2, 10.3, 10.8, 10.9 | 4.1, 5.1, 10.2 | Effective |
| King 2017- King 2020  USA  [73,74] | RCT | ECA | Carmen | Coach-like | Desktop computer | Lifestyle | Physical activity advice and support | TTM, SCT | 1.1, 1.2, 1.3, 1.4, 1.5, 1.7, 2.2, 2.3, 2.7, 3.3, 4.1, 5.1, 6.1, 8.2, 9.2, 10.1, 10.2, 10.3, 10.8 | 1.1, 1.2, 1.3, 1.4, 1.5, 1.7, 2.2, 2.3, 2.7, 3.1, 4.1, 5.1, 6.1, 8.2, 10.2, 10.3, 10.8 | Effective |
| Kowatsch 2021  Switzerland  [59] | Feasibility study | Avatar | Max | Coach-like | Smartphone app (adolescents)/ Desktop computer (HCP)/ SMS (parent) | Chronic disorders | Increase cognitive skills and behavioral skills in 10-15-year-olds with asthma | TPB, SDT, Technology Acceptance theories | 1.1, 1.2, 1.4, 1.7, 3.1, 3.3, 4.1, 5.1, 6.1, 6.2, 6.3, 7.1, 8.7, 10.2, 10.3, 12.1 | NA | NA |
| Kramer 2020  Switzerland  [75] | microRCTs | Avatar | Ally (Assistant to Lift your Level of activitY) | Coach-like | Smartphone app | Lifestyle | Increase and maintain physical activity | Not mentioned | 1.2, 1.4, 1.6, 2.2, 4.1, 7.1, 8.3, 10.2, 10.3 | 1.2, 1.4, 1.6, 2.2, 4.1 | Mixed |
| Krishnakumar 2021  India  [54] | Single group pretest-posttest trial | Not specified | Wellthy CARE mobile app | Coach-like | Smartphone app/ Desktop computer | Chronic disorders | Improve glycemic control for individuals with type 2 diabetes | Not mentioned | 1.2, 2.3, 2.4, 2.6, 2.7, 3.1, 3.3, 4.1, 6.1, 7.1, 8.1, 8.3, 9.1, 10.3, 11.1, 11.2, 12.3, 15.1 | NA | NA |
| Ly 2017  Sweden  [78] | Pilot study | No avatar | Shim | Coach-like | Smartphone app | Mental health | Improve psychological well-being and perceived stress | Not mentioned | 1.2, 1.5, 2.2, 3.3, 5.1, 5.4, 5.6, 9.1, 11.2, 12.2, 13.2, 13.4 | None | Effective |
| Maeda 2020  Japan  [76] | RCT | Avatar | Not specified | Not mentioned | Web based | Lifestyle | Fertility and preconception health education for women of reproductive age. | TTM | 4.1, 5.1, 5.2 | 4.1, 5.1, 5.2 | Effective |
| McDonald 2012  USA  [55] | Pilot study | Avatar | Not specified | HCP-like | Desktop computer | Chronic disorders | Communication training for patients with chronic pain | CAT | 4.1, 8.1 | 4.1 | No difference |
| McDonald 2013  USA  [56] | Pilot study | Avatar | Not specified | HCP-like | Desktop computer based | Chronic disorders | Communication training for patients with chronic pain | CAT | 4.1, 8.1 | 4.1 | Mixed |
| Oh 2020  Korea  [39] | RCT | Avatar | Todaki | Coach-like | Smartphone app | Mental health | CBT for individuals with panic disorder | Not mentioned | 2.3, 3.3, 4.1, 5.1, 7.1, 7.7, 8.1, 8.3, 11.2, 12.6, 13.2 | 4.1 | Mixed |
| Owens 2019  USA  [57] | Single group pretest-posttest trial | ECA | iDecide | HCP-like | Smartphone/ Tablet computer | Chronic disorders | Prostate cancer education to enhance informed decision making | UTAUT, TCML | 4.1, 8.1 | NA | NA |
| Perski 2019  UK  [11] | RCT | No avatar | Quit Coach | Coach-like | Smartphone app (iPhone) | Lifestyle | Smoking cessation | Not mentioned | 1.2, 1.3, 1.4, 1.5, 1.9, 2.2, 2.3, 3.1, 4.1, 5.1, 7.1, 8.3, 8.7, 9.2, 10.3, 10.10, 11.1, 11.3, 12.1, 12.3, 13.5 | 1.3, 1.4, 1.5, 1.9, 2.2, 2.3, 5.1, 10.3, 10.10, | Effective |
| Phillip 2020  France  [58] | Feasibility study | ECA | Louise | Coach-like | Smartphone app | Chronic disorders | Self-evaluation of sleep quality and self-management of insomnia. | Not mentioned | 2.4, 4.1, 7.1, 12.1, 12.3, 12.6 | NA | NA |
| Piao 2020  Korea  [77] | RCT | No avatar | Healthy Lifestyle Coaching Chatbot | Coach-like | Smartphone app | Lifestyle | Provide a health behavior intervention to support sustainable physical activity | Habit Formation Model | 1.1, 1.4, 6.2, 7.1, 8.3, 10.1, 10.3 | 1.1, 1.4, 7.1, 8.3, 10.1, 10.4 | Effective |
| Prochaska 2020  USA  [42] | Single group pretest-posttest trial | Avatar | W-SUDs (Woebot) | Coach-like | Smartphone app | Mental health | Support substance use disorder management | Not mentioned | 1.1, 2.1, 3.3, 7.1, 8.1, 8.3, 12.6 | NA | NA |
| So 2020  Japan  [43] | RCT | Not specified | GAMBOT | Coach-like | Smartphone app | Mental health | CBT for gambling disorder | Not mentioned | 1.1, 1.5, 2.2, 3.3, 4.1, 4.2, 6.2, 7.5, 12.1, 12.2, 12.3, 13.2 | 2.1 | No difference |
| Stephens 2019  USA  [60] | Feasibility study | Avatar | Tess | Coach-like | SMS | Chronic disorders | Weight management and prediabetes counseling in pediatric population. | Not mentioned | 1.1, 1.4, 1.5, 3.3, 4.1, 7.1, 8.3, 11.2 | NA | NA |
| Suganuma 2018  Japan [87] | Non-randomized comparison study | ECA | Sabori | Coach-like | Web based | Mental health | Deliver iCBT to prevent mental health issues | Not mentioned | 2.2, 2.3, 3.3 | None | Effective |
| Watson 2012  USA  [61] | RCT | ECA | Not specified | Coach-like | Desktop computer | Chronic disorders | Increase physical activity in overweight and obese individuals | Not mentioned | 1.1, 1.2, 2.3, 3.1, 3.3, 5.1, 10.3, 14.4 | 2.3 | Mixed |

**ART**: Antiretroviral therapy; **BBTPE**: Broaden and Build Theory of Positive Emotion; **CAT**: Communication accommodation theory; **CBT**: Cognitive Behavioral Therapy; **Con**: Control; **ECA**: Embodied conversational agent; **HAPA**: Health Action Process Approach; **HCP**: Healthcare provider; **IBS**: Inflammatory Bowel Syndrome; **Int**: Intervention; **MSM**: Men who have Sex with Men; **RCT**: Randomized controlled trial; **SCT**: Social Cognitive Theory; **SDT**: Self-Determination Theory; **SMS**: Short Message System; **TCML**: Theory of Cognitive Multimedia Learning; **TPB**: Theory of Planned Behavior; **TTM**: Transtheoretical Model; **UTAUT**: Unified Theory of Acceptance and Use of Technology
