# Supplementary material for: Conversational Agents in Health Care: Scoping Review of Their Behavior Change Techniques and Underpinning Theory
Source: J Med Internet Res. 2022 Oct 3;24(10):e39243. doi: 10.2196/39243 (PMC9577715; doi:10.2196/39243)
Supplement: Multimedia Appendix 4 [file jmir_v24i10e39243_app4.docx]

# Multimedia Appendix 4

## Use of BCTs according to the clinical domain

**Table S1**: Number of studies including each BCT, according to clinical domain

| **BC Techniques** | **Chronic disorder** | **Lifestyle** | **Mental Health** | ***p*** |
| --- | --- | --- | --- | --- |
| **# of BCTs** | **n (%)** | **n (%)** | **n (%)** |  |
| 15+ | 2 (14%) | 2 (14%) | 1 (5%) | 0.4695 |
| 11-15 | 3 (21%) | 2 (14%) | 4 (21%) |  |
| 6-10 | 6 (43%) | 9 (64%) | 7 (37%) |  |
| 1-5 | 3 (21%) | 1 (7%) | 7 (37%) |  |
| **BCT groups** |  | | | |
| **1. Goals & planning**  1.1 Goal setting (behaviour)  1.2 Problem solving  1.3 Goal setting (outcome)  1.4 Action planning  1.5 Review behaviour goal(s)  1.6 Discrepancy between current behaviour and   goal  1.7 Review outcome goal(s)  1.9 Commitment | **8 (57%)**  5 (36%)  6 (43%)  0 (0%)  3 (21%)  1 (7%)  0 (0%)  1 (7%)  0 (0%) | ***12 (86%)***  ***9 (64%)***  ***11 (79%)***  3 (21%)  ***9 (64%)***  ***6 (43%)***  2 (14%)  1 (7%)  2 (14%) | **13 (68%)**  7 (37%)  7 (37%)  0 (0%)  2 (11%)  6 (32%)  0 (0%)  0 (0%)  0 (0%) | 0.2897 |
| **2. Feedback and monitoring**  2.1 Monitoring of behaviour by others without   feedback  2.2 Feedback on behaviour  2.3 Self-monitoring of behaviour  2.4 Self-monitoring of outcome(s) of behaviour  2.5 Monitoring outcome(s) of behaviour by others   without feedback  2.6 Biofeedback  2.7 Feedback on outcome(s) of behaviour | **7 (50%)**  0 (0%)  2 (14%)  5 (36%)  3 (21%)  1 (7%)  4 (29%)  3 (21%) | **10 (71%)**  0 (0%)  ***7 (50%)***  ***9 (64%)***  0 (0%)  0 (0%)  0 (0%)  1 (7%) | ***17 (89%)***  4 (21%)  7 (37%)  6 (32%)  3 (16%)  0 (0%)  0 (0%)  0 (0%) | ***0.04689*** |
| **3. Social support**  3.1 Social support (unspecified)  3.2 Social support (practical)  3.3 Social support (emotional) | **7 (50%)**  3 (21%)  1 (7%)  7 (50%) | ***10 (71%)***  2 (14%)  1 (7%)  8 (57%) | **12 (63%)**  0 (0%)  0 (0%)  ***12 (63%)*** | 0.4994 |
| **4. Shaping knowledge**  4.1 Instruction on how to perform a behaviour  4.2 Information about antecedents  4.3 Re-attribution  4.4 Behavioural experiments | ***13 (93%)***  ***13 (93%)***  2 (14%)  1 (7%)  1 (7%) | **11 (79%)**  10 (71%)  1 (7%)  2 (14%)  1 (7%) | **10 (53%)**  9 (47%)  3 (16%)  2 (11%)  1 (5%) | ***0.0306*** |
| **5. Natural consequences**  5.1 Information about health consequences  5.2 Salience of consequences  5.3 Information about social and environmental   consequences  5.4 Monitoring of emotional consequences  5.5 Anticipated regret  5.6 Information about emotional consequences | **7 (50%)**  5 (36%)  1 (7%)  0 (0%)  1 (7%)  0 (0%)  1 (7%) | **7 (50%)**  7 (50%)  1 (7%)  0 (0%)  0 (0%)  0 (0%)  0 (0%) | **7 (37%)**  3 (16%)  0 (0%)  1 (5%)  3 (16%)  1 (5%)  3 (16%) | 0.6727 |
| **6. Comparison of behaviour**  6.1 Demonstration of the behaviour  6.2 Social comparison  6.3 Information about others’ approval | **5 (36%)**  4 (29%)  1 (7%)  2 (14%) | **3 (21%)**  2 (14%)  1 (7%)  0 (0%) | **2 (11%)**  1 (5%)  1 (5%)  0 (0%) | 0.2183 |
| **7. Associations**  7.1 Prompts/cues  7.5 Remove aversive stimulus  7.7 Exposure | **9 (64%)**  **8 (57%)**  0 (0%)  1 (7%) | **4 (29%)**  4 (29%)  0 (0%)  0 (0%) | **9 (47%)**  7 (37%)  1 (5%)  2 (11%) | 0.1661 |
| **8. Repetition and substitution**  8.1 Behavioural practice/ rehearsal  8.2 Behaviour substitution  8.3 Habit formation  8.6 Generalization of a target behaviour  8.7 Graded tasks | ***11 (79%)***  6 (43%)  0 (0%)  6 (43%)  0 (0%)  1 (7%) | **7 (50%)**  0 (0%)  3 (21%)  4 (29%)  0 (0%)  1 (7%) | **10 (53%)**  7 (37%)  1 (5%)  7 (37%)  1 (5%)  1 (5%) | 0.2219 |
| **9. Comparison of outcomes**  9.1 Credible source  9.2 Pros and cons  9.3 Comparative imagining of future outcomes | **2 (14%)**  2 (14%)  0 (0%)  0 (0%) | **5 (36%)**  2 (14%)  3 (21%)  0 (0%) | **4 (21%)**  2 (11%)  0 (0%)  2 (11%) | 0.492 |
| **10. Reward and threat**  10.1 Material incentive (behaviour)  10.2 Material reward (behaviour)  10.3 Non-specific reward  10.4 Social reward  10.6 Non-specific incentive  10.8 Incentive (outcome) – Includes positive   reinforcement  10.9 Self-reward  10.10 Reward (outcome) | **5 (36%)**  2 (14%)  1 (7%)  3 (21%)  0 (0%)  0 (0%)  0 (0%)  0 (0%)  0 (0%) | **8 (57%)**  2 (14%)  3 (21%)  **7 (50%)**  0 (0%)  1 (7%)  2 (14%)  1 (7%)  1 (7%) | **3 (16%)**  0 (0%)  0 (0%)  2 (11%)  2 (11%)  0 (0%)  0 (0%)  0 (0%)  0 (0%) | ***0.04743*** |
| **11. Regulation**  11.1 Pharmacological support  11.2 Reduce negative emotions  11.3 Conserving mental resources | **4 (29%)**  1 (7%)  4 (29%)  0 (0%) | **2 (14%)**  1 (7%)  1 (7%)  1 (7%) | **11 (58%)**  0 (0%)  **11 (58%)**  0 (0%) | 0.06849 |
| **12. Antecedents**  12.1 Restructuring the physical environment  12.2 Restructuring the social environment  12.3 Avoidance/ reducing exposure to cues for the   behaviour  12.6 Body changes | **5 (36%)**  2 (14%)  0 (0%)  2 (14%)  3 (21%) | **3 (21%)**  1 (7%)  1 (7%)  1 (7%)  1 (7%) | **7 (37%)**  2 (11%)  2 (11%)  1 (5%)  5 (26%) | 0.7261 |
| **13. Identity**  13.2 Framing/ reframing  13.4 Valued self-identity  13.5 Identity associated with changed behavior | **1 (7%)**  1 (7%)  0 (0%)  0 (0%) | **3 (21%)**  1 (7%)  1 (7%)  1 (7%) | **5 (26%)**  5 (26%)  1 (5%)  0 (0%) | 0.4257 |
| **14. Scheduled consequences**  14.4 Reward approximation | **1 (7%)**  1 (7%) | **0 (0%)**  0 (0%) | **0 (0%)**  0 (0%) | 1 |
| **15. Self-belief**  15.1 Verbal persuasion about capability  15.4 Self-talk | **3 (21%)**  3 (21%)  0 (0%) | **1 (7%)**  1 (7%)  0 (0%) | **2 (11%)**  1 (5%)  1 (5%) | 0.6382 |

*Test of significance for categorical variables was used in the assessments (chi-square or Fisher test
