# Supplementary material for: Conversational Agents in Health Care: Scoping Review of Their Behavior Change Techniques and Underpinning Theory
Source: J Med Internet Res. 2022 Oct 3;24(10):e39243. doi: 10.2196/39243 (PMC9577715; doi:10.2196/39243)
Supplement: Multimedia Appendix 5 [file jmir_v24i10e39243_app5.docx]

# Multimedia Appendix 5

## Frequent Itemset Mining (FIM)

BCTs frequently included together with Confidence larger than 0.90 and sorted by the support value, reported for all included studies and each clinical domain

| All studies | | | | Mental health | | |
| --- | --- | --- | --- | --- | --- | --- |
| Itemset  {bct4.1, bct8.1}  {bct4.1, bct7.1, bct8.3}  {bct1.1, bct1.5, bct3.3}  {bct1.2, bct1.4, bct4.1}  {bct3.3, bct4.1, bct11.2}  {bct1.1, bct1.2, bct5.1}  {bct1.2, bct2.3, bct10.3}  {bct2.3, bct4.1, bct7.1}  {bct1.1, bct1.2, bct2.3, bct5.1}  {bct1.1, bct1.2, bct3.3, bct5.1} | **Support**  0.255  0.234  0.213  0.213  0.213  0.191  0.170  0.170  0.149  0.149 | | | **Itemset**  **{**bct1.5, bct2.2, bct3.3**}**  **{**bct3.3, bct12.6**}**  **{**bct3.3, bct4.1, bct11.2**}**  **{**bct1.1, bct1.5, bct2.2, bct3.3**}**  **{**bct1.1, bct1.5, bct2.2**}**  **{**bct1.1, bct2.2, bct3.3**}**  **{**bct1.1, bct3.3, bct11.2**}**  **{**bct1.5, bct2.2, bct11.2**}**  **{**bct1.5, bct2.2, bct3.3, bct11.2**}**  **{**bct1.5, bct3.3, bct11.2**}** | **Support**  0.263  0.263  0.263  0.211  0.211  0.211  0.211  0.211  0.211  0.211 | |
| Lifestyle | | | **Chronic disorders** | | | |
| Itemset  {bct1.2, bct2.3}  {bct1.1, bct1.2, bct2.3}  {bct1.2, bct1.4, bct4.1}  {bct1.2, bct2.2}  {bct1.2, bct2.3, bct4.1}  {bct1.1, bct1.2, bct2.3, bct3.3}  {bct1.1, bct1.2, bct2.3, bct4.1}  {bct1.1, bct1.2, bct3.3}  {bct1.1, bct1.2, bct4.1}  {bct1.1, bct2.3, bct3.3} | | **Support**  0.643  0.571  0.500  0.500  0.500  0.429  0.429  0.429  0.429  0.429 | **Itemset**  **{**bct4.1**}**  **{**bct4.1, bct7.1**}**  **{**bct4.1, bct7.1, bct8.3**}**  **{**bct4.1, bct8.1**}**  **{**bct4.1, bct8.3**}**  **{**bct7.1, bct8.3**}**  **{**bct3.3, bct4.1, bct7.1**}**  **{**bct1.1, bct4.1, bct7.1**}**  **{**bct2.3, bct4.1, bct7.1, bct8.3**}**  **{**bct2.3, bct4.1, bct7.1**}** | | | **Support**  0.929  0.571  0.429  0.429  0.429  0.429  0.357  0.286  0.286  0.286 |
