# Supplementary material for: Conversational Agents in Health Care: Scoping Review of Their Behavior Change Techniques and Underpinning Theory
Source: J Med Internet Res. 2022 Oct 3;24(10):e39243. doi: 10.2196/39243 (PMC9577715; doi:10.2196/39243)
Supplement: Multimedia Appendix 6 [file jmir_v24i10e39243_app6.docx]

# Multimedia Appendix 6

# Use of BCTs according to the CA type

Figure S1 presents a summary of the BCTs used by experimental and comparison interventions according to CA type.

**
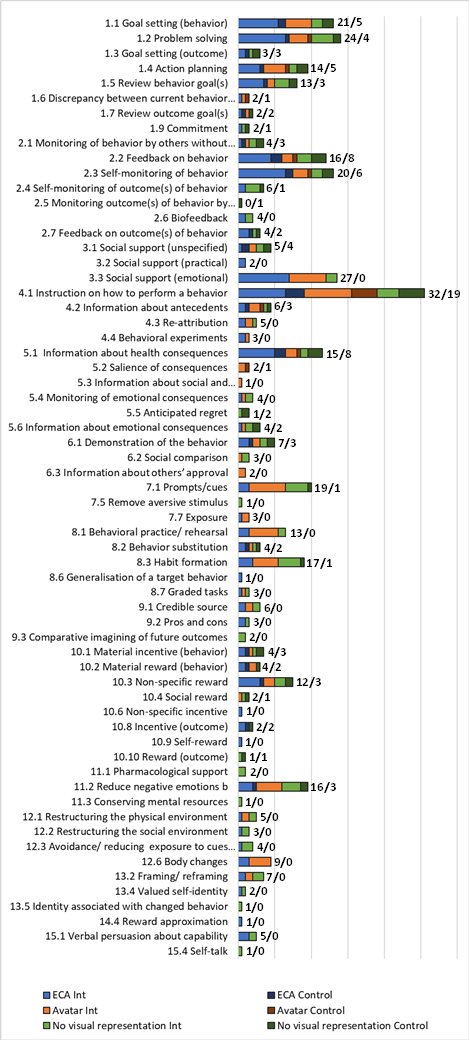
**

**Figure S1:** Number of studies including each BCT, according to conversational agent type

##### Embodied CA

ECAs were included in 20 studies (43%), including nine lifestyle change interventions (45%), six chronic disorder management interventions (30%), and five mental health interventions (25%). Most ECAs represented a female character (18/20, 90%)

ECA interventions incorporated 49 different BCTs. The most commonly used BCT was BCT 3.3 “Social support (emotional) (14/20, 70%), followed by BCT 1.2 “Problem solving”, BCT 2.3 “Self-monitoring of behaviour”, and BCT 4.1 “Instruction on how to perform a behaviour” found in 13 studies each (65%), BCT 1.1 “Goal setting (behaviour) incorporated in 11 studies (55%), and BCT 5.1 “Information about health consequences” used in 10 studies (50%).

##### CAs represented by an avatar

Avatars were the visual representation of CAs used in 15 studies, including six studies focusing on chronic disorder management (40%), six mental health interventions (40%) and three lifestyle change interventions (20%). Five mental health CAs included a non-human avatar, while the rest of CAs were represented by a human-like avatar.

Avatar CA interventions included a total of 38 BCTs. The most frequently incorporated BCT was 4.1 “Instruction on how to perform a behaviour” (13/15, 87%), followed by BCT 3.3 “Social support (emotional)” and BCT 7.1 ”Prompts/cues” included in 10 studies each (67%), and BCT 8.1 “Behavioural practice/rehearsal” included in 8/15 studies (53%).

##### CAs with non-specified or non-visual representation

Twelve CAs did not include a visual representation, or this was not evident from the description of the intervention. Most of the interventions included in this group belonged to the mental health domain (8/12, 67%), while there were two studies each from the chronic disorder management and lifestyle change domains (17% each).

A total of 47 BCTs were included in these interventions, however, no single BCT was predominant. Four BCTs were included in six studies (50%) each, including BCT 1.2 “Problem solving”, BCT 4.1 “Instruction on how to perform a behaviour”, BCT 7.1 ”Prompts/cues”, and BCT 8.3 “Habit formation”. Additionally, five studies (42%) included BCT 11.2 “Reduce negative emotions”, and four studies (33%) incorporated BCT 1.5 “Review behaviour goal(s)”, BCT 2.2 “Feedback on behaviour”, and BCT 2.4 “Self-monitoring of outcome(s) of behaviour".

Figure S2 presents a summary of the most commonly used BCTs according to CA type, and Table S1 presents a detailed summary of the number of studies including each BCT and BCT category, according to CA type.

**Figure S2**: Commonly used BCTs according to CA type

**Table S1**: Number of studies including each BCT, according to type of conversational agent

| **BCTs** | **ECA (n=20)**  **n (%)** | **Avatar (n=15)**  **n (%)** | **No representation (n=12), n (%)** |
| --- | --- | --- | --- |
| **1. Goals & planning**  1.1 Goal setting (behaviour)  1.2 Problem solving  1.3 Goal setting (outcome)  1.4 Action planning  1.5 Review behaviour goal(s)  1.6 Discrepancy between current behaviour and   goal  1.7 Review outcome goal(s)  1.9 Commitment | ***14 (70%)***  11 (55%)  13 (65%)  2 (10%)  6 (30%)  7 (35%)  1 (5%)  1 (5%)  1 (5%) | ***10 (67%)***  7 (47%)  5 (33%)  0 (0%)  6 (40%)  2 (13%)  1 (7%)  1 (7%)  0 (0%) | ***9 (75%)***  3 (25%)  6 (50%)  1 (8%)  2 (17%)  4 (33%)  0 (0%)  0 (0%)  1 (8%) |
| **2. Feedback and monitoring**  2.1 Monitoring of behaviour by others without   feedback  2.2 Feedback on behaviour  2.3 Self-monitoring of behaviour  2.4 Self-monitoring of outcome(s) of behaviour  2.5 Monitoring outcome(s) of behaviour by others   without feedback  2.6 Biofeedback  2.7 Feedback on outcome(s) of behaviour | ***16 (80%)***  1 (5%)  9 (45%)  13 (65%)  2 (10%)  0 (0%)  2 (10%)  3 (15%) | **7 (47%)**  1 (7%)  3 (20%)  4 (27%)  0 (0%)  0 (0%)  0 (0%)  0 (0%) | ***11 (92%)***  2 (17%)  4 (33%)  3 (25%)  4 (33%)  1 (8%)  2 (17%)  1 (8%) |
| **3. Social support**  3.1 Social support (unspecified)  3.2 Social support (practical)  3.3 Social support (emotional) | ***14 (70%)***  1 (5%)  2 (10%)  14 (70%) | ***11 (73%)***  2 (13%)  0 (0%)  10 (67%) | **4 (33%)**  2 (17%)  0 (0%)  3 (25%) |
| **4. Shaping knowledge**  4.1 Instruction on how to perform a behaviour  4.2 Information about antecedents  4.3 Re-attribution  4.4 Behavioural experiments | ***14 (70%)***  13 (65%)  2 (10%)  2 (10%)  2 (10%) | ***13 (87%)***  13 (87%)  3 (20%)  2 (13%)  1 (7%) | ***7 (58%)***  6 (50%)  1 (8%)  1 (8%)  0 (0%) |
| **5. Natural consequences**  5.1 Information about health consequences  5.2 Salience of consequences  5.3 Information about social and environmental   consequences  5.4 Monitoring of emotional consequences  5.5 Anticipated regret  5.6 Information about emotional consequences | ***11 (55%)***  10 (50%)  0 (0%)  0 (0%)  1(5%)  0 (0%)  1 (5%) | **6 (40%)**  3 (20%)  2 (13%)  1 (7%)  1 (7%)  0 (0%)  1 (7%) | **4 (33%)**  2 (17%)  0 (0%)  0 (0%)  2 (17%)  1 (8%)  2 (17%) |
| **6. Comparison of behaviour**  6.1 Demonstration of the behaviour  6.2 Social comparison  6.3 Information about others’ approval | **3 (15%)**  3 (15%)  0 (0%)  0 (0%) | **3 (20%)**  2 (13%)  1 (7%)  2 (13%) | **4 (33%)**  2 (17%)  2 (17%)  0 (0%) |
| **7. Associations**  7.1 Prompts/cues  7.5 Remove aversive stimulus  7.7 Exposure | **4 (20%)**  3 (15%)  0 (0%)  1 (5%) | ***11 (73%)***  10 (67%)  0 (0%)  2 (13%) | ***7 (58%)***  6 (50%)  1 (8%)  0 (0%) |
| **8. Repetition and substitution**  8.1 Behavioural practice/ rehearsal  8.2 Behaviour substitution  8.3 Habit formation  8.6 Generalization of a target behaviour  8.7 Graded tasks | **8 (40%)**  3 (15%)  2 (10%)  4 (20%)  1 (5%)  1 (5%) | ***13 (87%)***  8 (53%)  1 (7%)  7 (47%)  0 (0%)  1 (7%) | ***7 (58%)***  2 (17%)  1 (8%)  6 (50%)  0 (0%)  1 (8%) |
| **9. Comparison of outcomes**  9.1 Credible source  9.2 Pros and cons  9.3 Comparative imagining of future outcomes | **4 (20%)**  2 (10%)  2 (10%)  0 (0%) | **2 (13%)**  2 (13%)  0 (0%)  0 (0%) | **5 (42%)**  2 (17%)  1 (8%)  2 (17%) |
| **10. Reward and threat**  10.1 Material incentive (behaviour)  10.2 Material reward (behaviour)  10.3 Non-specific reward  10.4 Social reward  10.6 Non-specific incentive  10.8 Incentive (outcome) – Includes positive   reinforcement  10.9 Self-reward  10.10 Reward (outcome) | **8 (40%)**  2 (10%)  2 (10%)  6 (30%)  0 (0%)  1 (5%)  2 (10%)  1 (5%)  0 (0%) | **4 (27%)**  1 (7%)  2 (13%)  3 (20%)  1 (7%)  0 (0%)  0 (0%)  0 (0%)  0 (0%) | **4 (33%)**  1 (8%)  0 (0%)  3 (25%)  1 (8%)  0 (0%)  0 (0%)  0 (0%)  1 (8%) |
| **11. Regulation**  11.1 Pharmacological support  11.2 Reduce negative emotions  11.3 Conserving mental resources | **4 (20%)**  0 (0%)  4 (20%)  0 (0%) | **7 (47%)**  0 (0%)  7 (47%)  0 (0%) | **6 (50%)**  2 (17%)  5 (42%)  1 (8%) |
| **12. Antecedents**  12.1 Restructuring the physical environment  12.2 Restructuring the social environment  12.3 Avoidance/ reducing exposure to cues for the   behaviour  12.6 Body changes | **4 (20%)**  1 (5%)  1 (5%)  1 (5%)  3 (15%) | **7 (47%)**  2 (13%)  0 (0%)  0 (0%)  6 (40%) | **4 (33%)**  2 (17%)  2 (17%)  3 (25%)  0 (0%) |
| **13. Identity**  13.2 Framing/ reframing  13.4 Valued self-identity  13.5 Identity associated with changed behavior | **3 (15%)**  2 (10%)  1 (5%)  0 (0%) | **2 (13%)**  2 (13%)  0 (0%)  0 (0%) | **4 (33%)**  3 (25%)  1 (8%)  1 (8%) |
| **14. Scheduled consequences**  14.4 Reward approximation | **1 (5%)**  1 (5%) | **0 (0%)**  0 (0%) | **0 (0%)**  0 (0%) |
| **15. Self-belief**  15.1 Verbal persuasion about capability  15.4 Self-talk | **3 (15%)**  3 (15%)  0 (0%) | **0 (0%)**  0 (0%)  0 (0%) | **3 (25%)**  2 (17%)  1 (8%) |
